# Moving Biosurveillance Beyond Coded Data Using AI for Symptom Detection From Physician Notes: Retrospective Cohort Study

**DOI:** 10.2196/53367

**Published:** 2024-04-04

**Authors:** Andrew J McMurry, Amy R Zipursky, Alon Geva, Karen L Olson, James R Jones, Vladimir Ignatov, Timothy A Miller, Kenneth D Mandl

**Affiliations:** 1 Computational Health Informatics Program Boston Children's Hospital Boston, MA United States; 2 Department of Pediatrics Harvard Medical School Boston, MA United States; 3 Division of Pediatric Emergency Medicine Department of Pediatrics The Hospital for Sick Children Toronto, ON Canada; 4 Division of Critical Care Medicine Department of Anesthesiology, Critical Care, and Pain Medicine Boston Children's Hospital Boston, MA United States; 5 Department of Anaesthesia Harvard Medical School Boston, MA United States; 6 Department of Biomedical Informatics Harvard Medical School Boston, MA United States

**Keywords:** natural language processing, COVID-19, artificial intelligence, AI, public health, biosurveillance, surveillance, respiratory, infectious, pulmonary, SARS-CoV-2, symptom, symptoms, detect, detection, pipeline, pipelines, clinical note, clinical notes, documentation, emergency, urgent, pediatric, pediatrics, paediatric, paediatrics, child, children, youth, adolescent, adolescents, teen, teens, teenager, teenagers, diagnose, diagnosis, diagnostic, diagnostics

## Abstract

**Background:**

Real-time surveillance of emerging infectious diseases necessitates a dynamically evolving, computable case definition, which frequently incorporates symptom-related criteria. For symptom detection, both population health monitoring platforms and research initiatives primarily depend on structured data extracted from electronic health records.

**Objective:**

This study sought to validate and test an artificial intelligence (AI)–based natural language processing (NLP) pipeline for detecting COVID-19 symptoms from physician notes in pediatric patients. We specifically study patients presenting to the emergency department (ED) who can be sentinel cases in an outbreak.

**Methods:**

Subjects in this retrospective cohort study are patients who are 21 years of age and younger, who presented to a pediatric ED at a large academic children’s hospital between March 1, 2020, and May 31, 2022. The ED notes for all patients were processed with an NLP pipeline tuned to detect the mention of 11 COVID-19 symptoms based on Centers for Disease Control and Prevention (CDC) criteria. For a gold standard, 3 subject matter experts labeled 226 ED notes and had strong agreement (*F*_1_-score=0.986; positive predictive value [PPV]=0.972; and sensitivity=1.0). *F*_1_-score, PPV, and sensitivity were used to compare the performance of both NLP and the *International Classification of Diseases, 10th Revision* (ICD-10) coding to the gold standard chart review. As a formative use case, variations in symptom patterns were measured across SARS-CoV-2 variant eras.

**Results:**

There were 85,678 ED encounters during the study period, including 4% (n=3420) with patients with COVID-19. NLP was more accurate at identifying encounters with patients that had any of the COVID-19 symptoms (*F*_1_-score=0.796) than ICD-10 codes (*F*_1_-score =0.451). NLP accuracy was higher for positive symptoms (sensitivity=0.930) than ICD-10 (sensitivity=0.300). However, ICD-10 accuracy was higher for negative symptoms (specificity=0.994) than NLP (specificity=0.917). Congestion or runny nose showed the highest accuracy difference (NLP: *F*_1_-score=0.828 and ICD-10: *F*_1_-score=0.042). For encounters with patients with COVID-19, prevalence estimates of each NLP symptom differed across variant eras. Patients with COVID-19 were more likely to have each NLP symptom detected than patients without this disease. Effect sizes (odds ratios) varied across pandemic eras.

**Conclusions:**

This study establishes the value of AI-based NLP as a highly effective tool for real-time COVID-19 symptom detection in pediatric patients, outperforming traditional ICD-10 methods. It also reveals the evolving nature of symptom prevalence across different virus variants, underscoring the need for dynamic, technology-driven approaches in infectious disease surveillance.

## Introduction

Real-time emerging infection surveillance requires a case definition that often involves symptomatology. To detect symptoms, population health monitoring systems and research studies tend to largely rely on structured data from electronic health records, including the *International Classification of Diseases, 10th Revision* (ICD-10) codes [1]. However, symptoms are not diagnoses and, therefore, may not be consistently coded, leading to incorrect estimates of the prevalence of COVID-19 symptoms [2]. Natural language processing (NLP) of unstructured data from electronic health records has proven useful in recognizing COVID-19 symptoms and identifying additional signs and symptoms compared to structured data alone [3,4]. However, surveillance of COVID-19 symptoms is nuanced as symptoms have been shown to differ by variant eras [5,6] and by age, with pediatric patients generally experiencing milder symptoms [7]. For example, while loss of taste or smell was reported with early COVID-19 variants, it was less commonly reported during the Omicron wave and in younger patients who more frequently experience fever and cough [8-11]. Understanding symptom patterns in children during different COVID-19 variant eras is important. Early in the pandemic, the availability of molecular testing was extremely limited. The less severe course of infection and varying presentations may lead to under testing due to mild symptoms [12], potentially underestimating pediatric COVID-19 cases. Additionally, relatively asymptomatic children can still transmit the virus. Tailoring interventions based on age-specific manifestations contribute to effective control of virus transmission within communities.

We sought to validate and test an open-source artificial intelligence (AI)–based NLP pipeline that includes a large language model (LLM) to detect COVID-19 symptoms from physician notes. As a formative use case, we sought to illustrate how this pipeline could detect COVID-19 symptoms and differentiate symptom patterns across SARS-CoV-2 variant eras in pediatric patients. We specifically study patients presenting to the emergency department (ED) who can be sentinel cases in an outbreak.

## Methods

### Study Design and Setting

This was a retrospective cohort study of all patients up to 21 years of age presenting to the ED of a large, free-standing, university-affiliated, pediatric hospital between March 1, 2020, and May 31, 2022.

### Ethical Considerations

The Boston Children’s Hospital Committee on Clinical Investigation performed ethical, privacy, and confidentiality reviews of the study and found it to be exempt from human subjects oversight. A waiver of consent was obtained to cover the targeted extraction and secure review of clinical notes by approved study personnel in protected environments within the hospital firewall.

### Study Variables

The main dependent variables were a set of 11 COVID-19 symptoms based on Centers for Disease Control and Prevention (CDC) criteria [13]—fever or chills, cough, shortness of breath or difficulty breathing, fatigue, muscle or body aches, headache, new loss of taste or smell, sore throat, congestion or runny nose, nausea or vomiting, and diarrhea. We identified these symptoms by both NLP and ICD-10 codes. For the formative use case, the study period was divided into 3 variant eras defined using Massachusetts COVID-19 data from Covariant [14]. The pre-Delta era was from March 1, 2020, to June 20, 2021; the Delta era was from June 21, 2021, to December 19, 2021; and the Omicron era was from December 20, 2021, onward. A diagnosis of COVID-19 was defined as a positive SARS-CoV-2 polymerase chain reaction (PCR) test or the presence of ICD-10 code U07.1 for COVID-19 during the same ED encounter in which symptoms were evaluated.

### AI/NLP Pipeline Development

A total of 3 reviewers reached a consensus on a symptom concept dictionary [15] to capture each of the 11 COVID-19 symptoms. They relied on the Unified Medical Language System [16], which has a nearly comprehensive list of symptom descriptors [17], including SNOMED (SNOMED International) coded clinical terms [18], ICD-10 codes for administrative billing, abbreviations, and common language for patients [19]. The open-source and free Apache cTAKES (Apache Software Foundation) NLP pipeline was tuned to recognize and extract coded concepts for positive symptom mentions (based on the dictionary) from physician notes [20]. Apache cTAKES uses a NegEx algorithm which can help address negation [20-23]. To further address negation, we incorporated an LLM, Bidirectional Encoder Representations from Transformers, that was fine-tuned for negation classification on clinical text [24,25].

### Gold Standard

A total of 2 reviewers established a gold standard by manually reviewing physician ED notes. After all notes were labeled by the cTAKES pipeline, a test set of 226 ED notes was loaded into Label Studio [26], an open-source application for ground truth labeling. These notes were from patients both with and without COVID-19 and were selected to ensure that each of the 11 symptoms was mentioned in at least 30 ED notes. Some notes mentioned more than 1 symptom. Using an annotation guide (Multimedia Appendix 1), 2 reviewers, who were masked from the terms identified by the NLP pipeline for note selection, each labeled 113 notes for mention of the 11 COVID-19 symptoms. As per the guide, only symptoms relevant to the present illness were considered positive mentions. Symptoms were not considered positive mentions if stated as past medical history, family history, social history, or an indication for a medication unrelated to the encounter.

### Interrater Reliability

The *F*_1_-score was used to assess consistency in manual chart review. The *F*_1_-score is the balance of sensitivity and positive predictive value (PPV) [27]. It was computed by comparing the annotations of each of the 2 initial reviewers to those of a third reviewer, who independently labeled a subset (56/226, 25%) of notes annotated by the other reviewers. The choice of *F*_1_-score as the metric for agreement was informed by the observed high frequency of true negative annotations when they were assigned by chance [20,27,28]. Reliability analyses used Python (version 3.10; Python Software Foundation).

### AI/NLP and ICD-10 Accuracy

Accuracy measures of the true symptom percentages in the test set for each symptom included *F*_1_-score, PPV, sensitivity, and specificity [29,30].

### Formative Use Case

The impact of pandemic variant era on COVID-19 symptomatology was examined. Descriptive statistics were used to characterize patients presenting to the ED during each pandemic era. The percentage of patients in the ED with symptoms of COVID-19 was assessed in separate analyses for each symptom using chi-square analyses of 3×2 tables (pandemic era × symptom presence or absence) with α set at .05. Post hoc chi-square tests were used to compare each pandemic era with all others using a Bonferroni adjusted α of .017. To assess the effect of pandemic era, COVID-19 status, and the interaction of these variables on whether or not a patient had each symptom, logistic regression was used in separate analyses for each symptom. Bonferroni adjusted confidence limits were used for post hoc analyses. If the interaction term was not significant, the main effects of COVID-19 and variant era were reported. Data were analyzed using SAS (version 9.4; SAS Institute Inc).

## Results

### Study Population

There were 59,173 unique patients with 85,678 ED encounters during the study period. For each ED encounter, there was 1 final physician ED note that aggregated all ED physician documentation. Characteristics of the entire study cohort and variant-specific cohorts are summarized in Table 1. A patient could appear in the cohort more than once if they had multiple ED encounters.

**Table 1:** Characteristics of patients at emergency department encounters.

Characteristics			Total (n=85,678), n (%)		Pre-Delta (n=38,985), n (%)		Delta (n=24,432), n (%)		Omicron (n=22,261), n (%)
**Age range (years)**									
	<5	36,835 (43.0)		15,403 (39.5)		11,749 (48.1)		9683 (43.5)	
	≥5	48,843 (57.0)		23,582 (60.5)		12,683 (51.9)		12,578 (56.5)	
**Sex**									
	Female	40,250 (47.0)		18,659 (47.9)		11,236 (46.0)		10,355 (46.5)	
	Male	45,428 (53.0)		20,326 (52.1)		13,196 (54.0)		11,906 (53.5)	
**Race**									
	American Indian	147 (0.2)		64 (0.2)		54 (0.2)		29 (0.1)	
	Asian	3244 (3.8)		1457 (3.7)		949 (3.9)		838 (3.8)	
	African American	13,354 (15.6)		6007 (15.4)		3943 (16.1)		3404 (15.3)	
	Pacific Islander	81 (0.1)		28 (0.1)		24 (0.1)		29 (0.1)	
	White	34,186 (39.9)		16,990 (43.6)		9093 (37.2)		8103 (36.4)	
	Not identified	34,666 (40.4)		14,439 (37.0)		10,369 (42.4)		9858 (44.2)	
**COVID-19 classification method**									
	COVID-19 diagnosis	3420 (4.0)		854 (2.2)		500 (2.0)		2066 (9.3)	
	PCR^a^ positive	2167 (2.5)		518 (1.3)		294 (1.2)		1355 (6.1)	
	ICD-10^b^ code	3305 (3.9)		820 (2.1)		458 (1.9)		2027 (9.1)	

^a^PCR: polymerase chain reaction.

^b^ICD-10: International Classification of Diseases, 10th Revision.

### Interrater Reliability

High consistency was demonstrated between reviewer 3, who labeled a subset of notes, and both reviewers 1 and 2, who each labeled half of the notes chosen to establish the gold standard. The *F*_1_-scores for the 2 reviewers were 0.988 and 0.984, respectively. The PPV was 0.976 and 0.968 and sensitivity was 1.0 for both.

### AI or NLP ICD-10 Accuracy

As shown in Table 2, the *F*_1_-score for NLP was higher and thus more accurate at identifying encounters in the test set with patients that had any of the COVID-19 symptoms than ICD-10. NLP also had higher *F*_1_-score for each individual symptom. In addition, NLP sensitivity of true positive symptoms was higher than ICD-10. However, NLP accuracy of true negative symptoms (specificity) was somewhat lower compared to ICD-10.

**Table 2:** Accuracy of COVID-19 symptom monitoring using NLP^a^ and ICD-10^b^ in the test set.

Symptom	*F*_1_-score^c^		PPV^d^		Sensitivity		Specificity		
	NLP, n	ICD-10, n	NLP, n	ICD-10, n	NLP, n	ICD-10, n	NLP, n	ICD-10, n	
Any COVID-19 symptom	0.796	0.451	0.696	0.906	0.930	0.300	0.917	0.994	
Congestion or runny nose	0.828	0.042	0.788	1.000	0.872	0.021	0.938	1.000	
Cough	0.914	0.541	0.841	0.952	1.000	0.377	0.942	0.994	
Diarrhea	0.629	0.474	0.489	0.692	0.880	0.360	0.884	0.980	
Fatigue	0.817	0.057	0.744	0.333	0.906	0.031	0.948	0.990	
Fever or chills	0.864	0.700	0.768	0.977	0.987	0.545	0.844	0.993	
Headache	0.744	0.566	0.667	1.000	0.842	0.395	0.914	1.000	
Loss of taste or smell	0.667	0.167	0.500	1.000	1.000	0.091	0.948	1.000	
Muscle or body aches	0.723	0.211	0.567	1.000	1.000	0.118	0.937	1.000	
Nausea or vomiting	0.820	0.535	0.722	0.885	0.950	0.383	0.866	0.982	
Shortness of breath or difficulty breathing	0.685	0.400	0.595	0.889	0.806	0.258	0.912	0.995	
Sore throat	0.774	0.207	0.649	0.750	0.960	0.120	0.935	0.995	

^a^NLP: natural language processing.

^b^ICD-10: International Classification of Diseases, 10th Revision.

^c^*F*_1_-score: accuracy measure balancing PPV and sensitivity.

^d^PPV: positive predictive value.

The 2 most prevalent symptoms, cough and fever, had sensitivity scores for NLP that were among the highest of the symptoms, and much higher than those for ICD-10 codes. The greatest discrepancy between NLP and ICD-10 *F*_1_-scores was for congestion or runny nose. The smallest difference was for diarrhea.

### Formative Use Case

#### Prevalence of Symptoms Over Time

**Figure 1:**
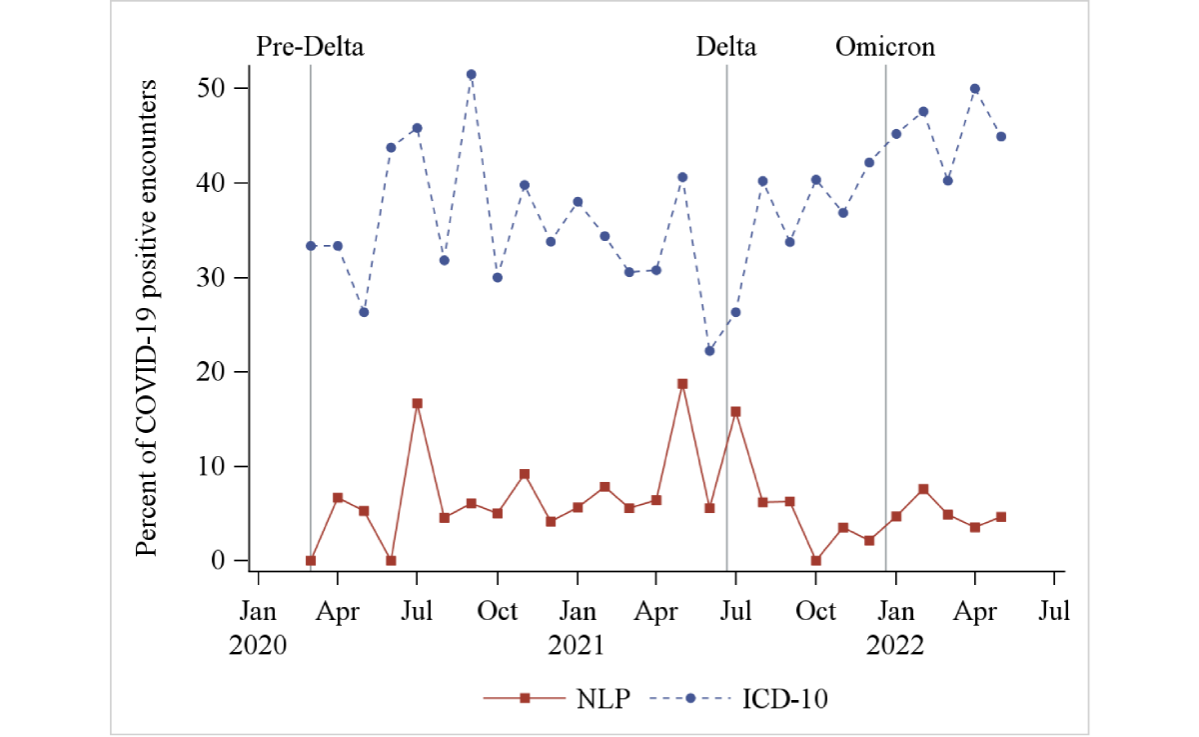
The percentage of encounters with patients with COVID-19 presenting to the emergency department each month with no symptoms detected, as measured using NLP and ICD-10. ICD-10: International Classification of Diseases, 10th Revision; NLP: natural language processing.

The percentage of ED encounters with patients with COVID-19 who had symptoms was estimated using the NLP pipeline and ICD-10 codes. As shown in Figure 1, during each month of the study, the percentage of encounters with no symptoms detected was much lower using NLP compared to ICD-10. Using NLP, the range was from 0% to 19% of encounters (mean 6%, SD 4%), while with ICD-10, the range was 22% to 52% (mean 38%, SD 7%).

**Figure 2:**
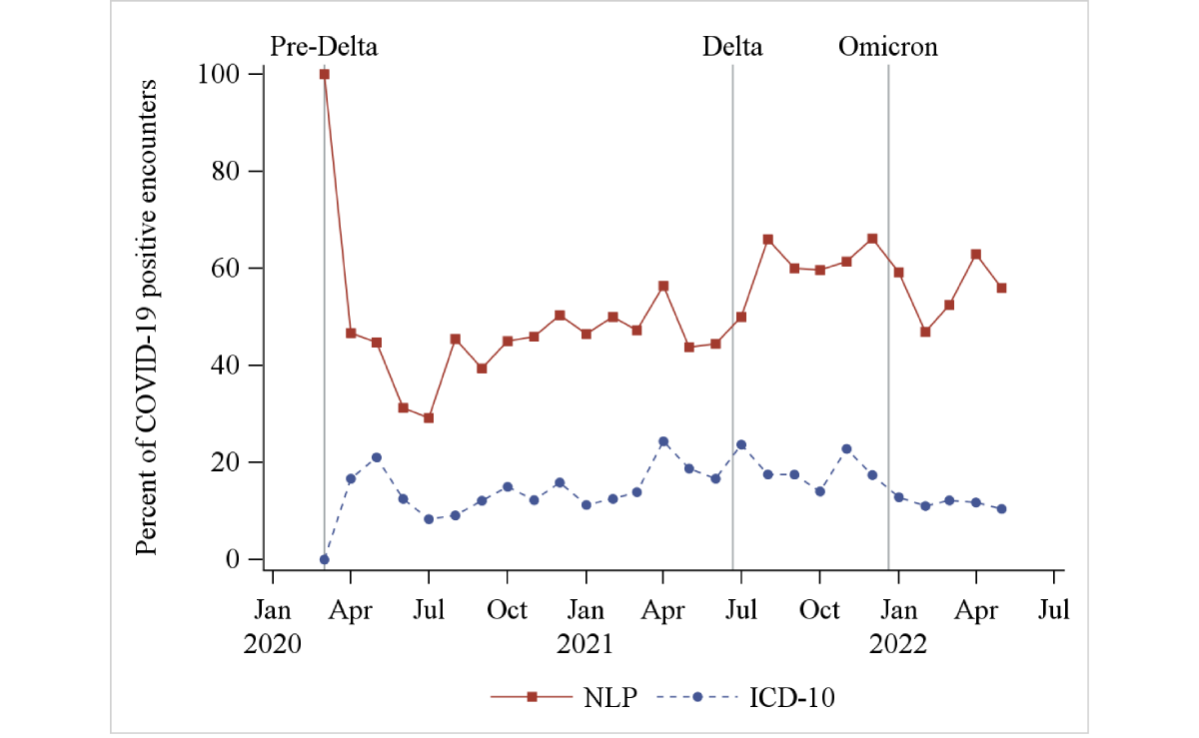
The percentage of encounters with patients with COVID-19 presenting to the emergency department each month with cough, as measured using NLP and ICD-10. ICD-10: International Classification of Diseases, 10th Revision; NLP: natural language processing.

**Figure 3:**
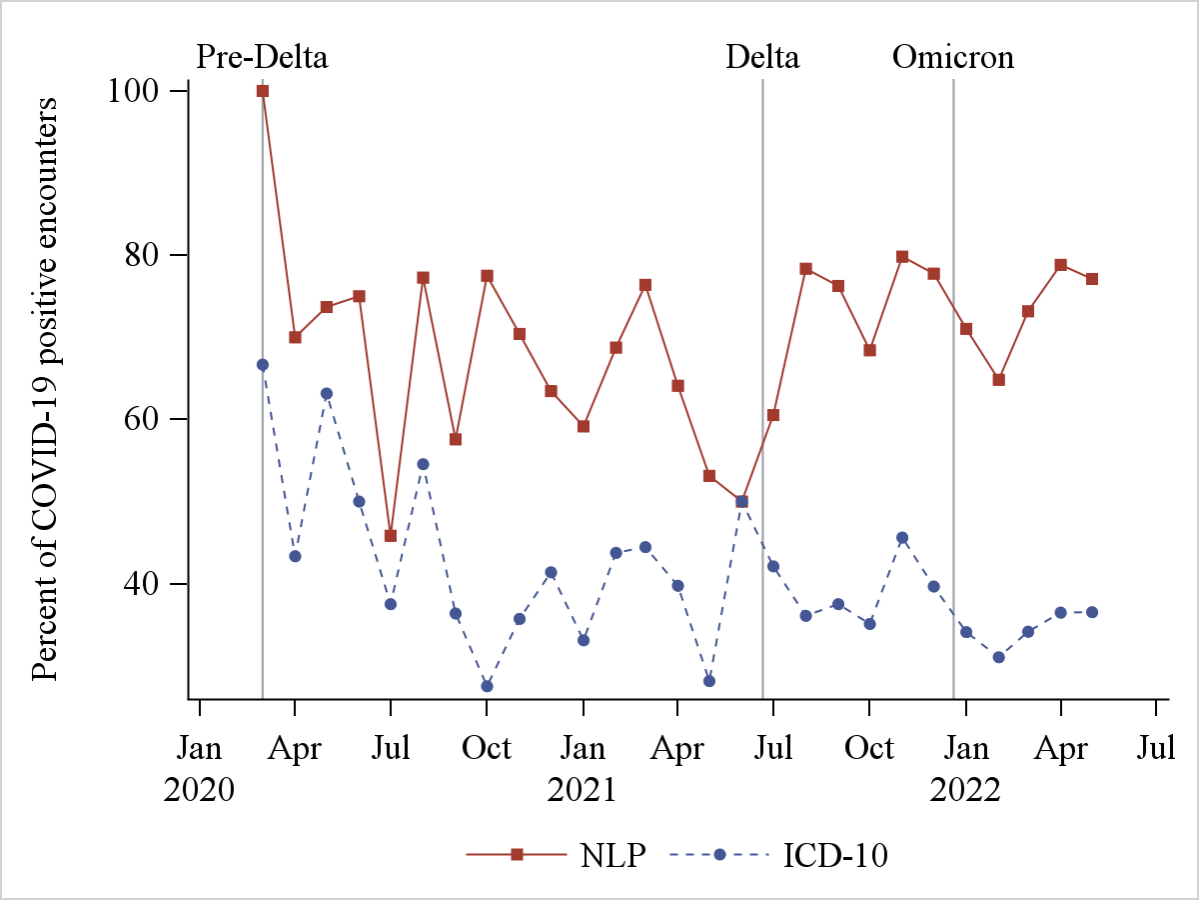
The percentage of encounters with patients with COVID-19 presenting to the emergency department each month with fever, as measured using NLP and ICD-10. ICD-10: International Classification of Diseases, 10th Revision; NLP: natural language processing.

The percentage of encounters with patients with COVID-19 who presented with each symptom by month was higher using NLP than ICD-10 (Multimedia Appendix 2). The 2 most common symptoms, cough and fever, are shown in Figures 2 and 3. On average, cough was identified during 52% (SD 13%) of the encounters each month using NLP, but only 15% (SD 5%) using ICD-10. On average, fever characterized 70% (SD 11%) of encounters using NLP, but 41% (SD 9%) using ICD-10.

Using ICD-10, there were many months where individual symptoms were not detected. Of the 27 study months, loss of taste or smell was not detected using ICD-10 during 24 months, nor were muscle or body aches during 13 months. A total of 3 more symptoms had at least 3 consecutive months where each was not detected using ICD-10. These were congestion or runny nose (9 total months, not all consecutive), sore throat (8 months), and fatigue (7 months). Sporadic months without detection using ICD-10 were observed for headache (5 months), diarrhea (2 months), cough (1 month), and nausea or vomiting (1 month). Using NLP, sporadic months without detection were observed for just 2 symptoms, loss of taste or smell (6 months) and sore throat (2 months).

#### Prevalence of Symptoms Across Variant Eras

The prevalence estimates of symptoms across variant eras for encounters with patients with COVID-19 differed for each symptom identified by NLP, except for nausea or vomiting and sore throat (Table 3). Post hoc analyses revealed several patterns. New loss of taste or smell was the only symptom that varied across all 3 eras. It was most common in the pre-Delta era, followed by the Delta era, and then the Omicron era. Congestion or runny nose, cough, and fever or chills were more common during the Delta and Omicron era than during the pre-Delta era, but the Delta era did not differ from the Omicron era. Muscle or body aches were more common during the pre-Delta era than both the Delta and Omicron eras, but the Delta era did not differ from the Omicron era. Diarrhea, fatigue, headache, and shortness of breath were more common during the pre-Delta era than the Omicron era but were not different than the Delta era, and the Delta era did not differ from the Omicron era. Nausea or vomiting and sore throat did not differ by variant era. The chi-square results are in Multimedia Appendix 3.

**Table 3:** Prevalence estimates of symptoms using natural language processing by variant era for emergency department encounters with patients with COVID-19.

Symptom	Pre-Delta era (n=854), n (%)	Delta era (n=500), n (%)	Omicron era (n=2066), n (%)	*P* value
Congestion or runny nose	250 (29.3)^a^	186 (37.2)^b^	742 (35.9)^b^	.001
Cough	402 (47.1)^a^	309 (61.8)^b^	1223 (59.2)^b^	<.001
Diarrhea	188 (22.0)^a^	92 (18.4)^a,b^	317 (15.4)^b^	<.001
Fatigue	129 (15.1)^a^	72 (14.4)^a,b^	228 (11.0)^b^	.004
Fever or chills	561 (65.7)^a^	376 (75.2)^b^	1525 (73.8)^b^	<.001
Headache	185 (21.7)^a^	92 (18.4)^a,b^	301 (14.6)^b^	<.001
Muscle or body aches	110 (12.9)^a^	39 (7.8)^b^	164 (7.9)^b^	<.001
Nausea or vomiting	297 (34.8)	170 (34.0)	709 (34.3)	.95
New loss of taste or smell	57 (6.7)^a^	9 (1.8)^b^	9 (0.4)^c^	<.001
Shortness of breath or difficulty breathing	182 (21.3)^a^	84 (16.8)^a,b^	311 (15.1)^b^	<.001
Sore throat	125 (14.6)	83 (16.6)	319 (15.4)	.63

^a,b,c^Variant eras with the same superscript across a row did not differ in post hoc analyses.

#### Symptoms by COVID-19 Status and Variant Era

The interaction of COVID-19 status and variant era on the presence of each symptom is shown in Table 4. However, because the interaction was not significant for 2 symptoms, fever and chills, and sore throat, the main effects for COVID-19 status are shown for both (*P*<.001). The odds ratios (ORs) indicate that patients with COVID-19 were more likely to have each of these 2 symptoms than patients without this disease. These symptoms were also more likely to occur during the Delta and Omicron era than during the pre-Delta era. For the remaining symptoms, the interaction term was significant and the ORs in each variant era are shown in the table. The ORs comparing patients with COVID-19 to those without the disease differed among the variant eras. Several patterns were observed. Patients with COVID-19 were more likely to exhibit each of the symptoms of congestion or runny nose, cough, fatigue, headache, muscle or body aches, new loss of taste or smell, or shortness of breath or difficulty breathing. However, effect sizes (ORs) differed among pandemic eras. For diarrhea, this symptom was more likely for patients with COVID-19 in the pre-Delta and Delta eras, but not during the Omicron era. And nausea was more likely only in the pre-Delta era. Significant ORs ranged in size from 1.3 to 26.7 (mean 4.6, SD 5.3). The logistic regression results are in Multimedia Appendix 4.

**Table 4:** Effect of COVID-19 status and variant era on the presence of each symptom detected using natural language processing.

Symptom and pandemic variant era			Odds ratio^a^ (95% CL^b^)		Interaction^c^ *P* value^d^	
**Congestion or runny nose**						*<.001*
	Pre-Delta	3.62 (3.11-4.21)				
	Delta	2.27 (1.89-2.72)				
	Omicron	2.46 (2.23-2.71)				
**Cough**						*<.001*
	Pre-Delta	4.84 (4.22-5.55)				
	Delta	3.64 (3.03-4.37)				
	Omicron	3.54 (3.23-3.88)				
**Diarrhea**						*<.001*
	Pre-Delta	2.23 (1.89-2.63)				
	Delta	1.42 (1.13-1.79)				
	Omicron	1.05 (0.92-1.19)				
**Fatigue**						*.01*
	Pre-Delta	3.22 (2.65-3.90)				
	Delta	3.42 (2.64-4.42)				
	Omicron	2.36 (2.03-2.75)				
Fever or chills			4.82 (4.46-5.21)		.66	
**Headache**						*<.001*
	Pre-Delta	2.33 (1.98-2.76)				
	Delta	2.09 (1.66-2.63)				
	Omicron	1.52 (1.33-1.73)				
**Muscle or body aches**						*.006*
	Pre-Delta	5.96 (4.83-7.36)				
	Delta	4.75 (3.38-6.67)				
	Omicron	3.78 (3.14-4.55)				
**Nausea or vomiting**						*.006*
	Pre-Delta	1.30 (1.13-1.50)				
	Delta	1.03 (0.86-1.25)				
	Omicron	0.98 (0.89-1.08)				
**New loss of taste or smell**						*.049*
	Pre-Delta	26.66 (19.13-37.14)				
	Delta	11.83 (5.68-24.65)				
	Omicron	11.04 (4.25-28.64)				
**Shortness of breath or difficulty breathing**						*<.001*
	Pre-Delta	2.62 (2.22-3.10)				
	Delta	1.70 (1.34-2.16)				
	Omicron	1.57 (1.38-1.79)				
Sore throat			2.45 (2.22-2.70)		.27	

^a^Odds ratios compare patients with COVID-19 at an ED encounter to patients without the disease.

^b^CL: Bonferroni adjusted confidence limits in post hoc analyses.

^c^If the interaction term was significant, the effect of COVID-19 during each variant era is shown. Otherwise, the effect for COVID-19 is shown.

^d^Type 3 test of the interaction term (variant era × COVID-19) in a logistic regression analysis.

## Discussion

### Principal Findings

We find evidence that AI-based NLP of physician notes is a superior method for capturing patient symptoms for real-time biosurveillance than reliance on traditional approaches using ICD-10. NLP was more sensitive than ICD-10 codes in identifying symptoms and some symptoms could only be detected using NLP. As a form of internal validation, the symptoms identified by the CDC as associated with COVID-19 were more common in patients with than without this disease.

### Comparison With Prior Work

The study was also able to capture a nuanced picture of symptom prevalence and odds across different SARS-CoV-2 variant eras. Consistent with previous literature, symptom patterns changed over time as new variants emerged. Variants may present with differences in symptomatology as a result of a number of factors including differences in mutations in spike proteins, receptor binding, and ability to escape host antibodies [31]. As has been previously reported [11,32-35], we found that fever or chills were the most common COVID-19 symptom across the variants. In our cohort, shortness of breath was less common during the Omicron era than during the pre-Delta era. The Omicron variant has less of an ability to replicate in the lungs compared to the bronchi, which may explain why this symptom became less common [36]. Studies have reported sore throat as a common symptom in the Omicron era, but we did not observe a significant difference across eras [8,9]. It is possible that we did not see a higher percentage of sore throats in the Omicron era because it may be more challenging for pediatric patients to describe this symptom. One study found that sore throat was observed more often in those of 5-20 years of age compared to those of 0-4 years of age [8]. Similarly, a study reported that sore throat was more common in those greater than or equal to 13 years of age in the Omicron era compared to the Delta era [37]. In our study cohort, approximately half of the patients were younger than 5 years of age. As children this age may not be able to describe their symptoms well, symptoms that are also signs, such as fever or cough, might be more commonly documented in physician notes than symptoms such as sore throat. New loss of taste or smell was most common in the pre-Delta era, followed by the Delta era and then the Omicron era in this study. This symptom has been reported less commonly in the Omicron era [8,9]. Studies have postulated that patients with the Omicron variant are less likely to present with loss of taste or smell as this variant has less penetration of the mucus layer and therefore, may be less likely to infect the olfactory epithelium [38].

### Limitations

There were important limitations in our use of NLP. The NLP pipeline was tested with a set of notes where some symptoms were more frequent in the test set (eg, loss of taste or smell) than in the formative use case. This was done to have sufficient data to evaluate the symptom pipeline. The NLP pipeline does not account for vital signs and so fever may not have been detected with the pipeline if it was documented in a patient’s vital signs rather than the clinical text. The cTAKES tool in the pipeline lacks the temporal context to ascertain if the mention of a symptom in a note is a new symptom or a prior symptom. We modified our technique because of this but nevertheless may have overestimated the prevalence of symptoms in our study. Future work will involve filtering by note section so that certain components of a note like past medical history are not included. We used 2 techniques to recognize negation, but some negated symptoms (eg, “patient had no cough”) were still captured as positive symptom mentions leading to a possible overestimation of symptom prevalence. Finally, this NLP pipeline did involve substantial preprocessing. We plan to evaluate the implementation of Generative Pre-trained Transformer (GPT) for this task. GPT-4 was able to extract COVID-19 symptoms in a recent study [39] and it may limit the need for preprocessing.

Our formative study had some limitations. First, we examined COVID-19 symptoms in patients presenting to a single urban pediatric ED. Patients presenting to outpatient settings, who likely had milder symptoms, were not included and our results may reflect patients with more severe symptoms. And because the setting was a single site, results may not generalize to other EDs. Second, we defined COVID-19 status as positive if a patient had a PCR positive test for COVID-19 or an appropriate ICD-10 code at the ED encounter. Patients who were COVID-19 positive on a test at home or at an outside center may not have been captured by this definition even if they presented to the ED with COVID-19 [40]. Additionally, symptoms may have differed across variant eras as a result of COVID-19 vaccinations or previous infections rather than variant differences. Literature in adults shows that vaccination is associated with a decrease in systemic symptoms [41]. The United States Food and Drug Administration authorized the use of the COVID-19 vaccine in October 2021, during the Delta era and prior to the Omicron era, for children 5-11 years of age [42]. Vaccination rates for pediatric patients vary by age group in Massachusetts, as of April 3, 2023, of those 0-19 years of age, 3% to 57% have received a primary series but have not been boosted, and 3% to 18% have been boosted since September 1, 2022 [43]. As such, some patients in the Delta and Omicron eras may have been vaccinated or had previous COVID-19 infections [44].

### Conclusions

In an era where rapid and accurate infectious disease surveillance is crucial, this study underscores the transformative potential of AI-based NLP for real-time symptom detection, significantly outperforming traditional methods such as ICD-10 coding. The dynamic adaptability of NLP technology allows for the nuanced capture of evolving symptomatology across different virus variants, offering a more responsive and precise tool kit for biosurveillance efforts. Its integration into existing health care infrastructure could be a game changer, elevating our capabilities to monitor, understand, and ultimately control the spread of emerging infectious diseases.

## Appendix

Multimedia Appendix 1 COVID-19 symptoms annotation guide.

Multimedia Appendix 2 Detection of COVID-19 symptoms using NLP and ICD-10 by month for emergency department encounters with patients with COVID-19. ICD-10: International Classification of Diseases, 10th Revision; NLP: natural language processing.

Multimedia Appendix 3 The chi-square analysis of COVID-19 symptom prevalence by pandemic variant era for emergency department encounters with patients with COVID-19, symptoms were detected using NLP. NLP: natural language processing.

Multimedia Appendix 4 Logistic regression analysis of the effect of COVID-19 status, pandemic variant era, and their interaction on symptom status for ED encounters, symptoms were detected using NLP. ED: emergency department; NLP: natural language processing.

Multimedia Appendix 5 Data files for the time series figures, the chi-square analysis of symptom prevalence, and the logistic regression analysis of the effects of COVID-19 status and pandemic variant era on symptom status.

## Abbreviations

AI: artificial intelligence
CDC: Centers for Disease Control and Prevention
ED: emergency department
GPT: Generative Pre-trained Transformer
ICD-10: International Classification of Diseases, 10th Revision
LLM: large language model
NLP: natural language processing
OR: odds ratio
PCR: polymerase chain reaction
PPV: positive predictive value

## Data Availability

All data analyzed during this study for the formative use case are in Multimedia Appendix 5 of this published article.
