# Supplementary material for: Moving Biosurveillance Beyond Coded Data Using AI for Symptom Detection From Physician Notes: Retrospective Cohort Study
Source: J Med Internet Res. 2024 Apr 4;26:e53367. doi: 10.2196/53367 (PMC11027052; doi:10.2196/53367)
Supplement: Multimedia Appendix 1 [file jmir_v26i1e53367_app1.pdf]

Multimedia Appendix 1. COVID-19 Symptom Annotation Guide.

Supplementary information for:

Moving Biosurveillance Beyond Coded Data Using AI for Symptom Detection from Physician Notes: Retrospective Cohort Study

Andrew J. McMurry,[1,2] Amy R. Zipursky,[1,3] Alon Geva,[1,4,5] Karen L. Olson,[1,2] James R. Jones,[1,2] Vlad Ignatov,[1] Timothy A. Miller,[1,2] Kenneth D. Mandl[1,2,6,*]

[1] Computational Health Informatics Program, Boston Children's Hospital, Boston, Massachusetts, United States of America
[2] Department of Pediatrics, Harvard Medical School, Boston, Massachusetts, United States of America
[3] Division of Emergency Medicine, The Hospital for Sick Children, Toronto, Ontario, Canada
[4] Division of Critical Care Medicine, Department of Anesthesiology, Critical Care, and Pain Medicine, Boston Children's Hospital, Boston, Massachusetts, United States of America
[5] Department of Anaesthesia, Harvard Medical School, Boston, MA, USA
[6] Department of Biomedical Informatics, Harvard Medical School, Boston, Massachusetts, United States of America

*Corresponding author
E-mail: kenneth_mandl@harvard.edu (KDM)

COVID-19 Symptom Annotation Guide

Three expert reviewers reached consensus on three criteria for annotating positive mentions of COVID-19 symptoms in ED physician notes. The three criteria are applied in series (1) encounter time criteria; (2) ED note section criteria; and (3) symptom specific criteria.

Criteria 1: Encounter time. Symptoms must be relevant to the present ED encounter either as the reason for visit, documented symptom, or observed sign.

Encounter Time	Include	Exclude
Prior to encounter	Chief complaint or patient reason for visit	Symptoms recorded for an illness or condition unrelated to the current encounter
Present ED encounter	Yes	Symptoms recorded for an illness or condition unrelated to the current encounter
Future encounter	Never	Always

Criteria 2: ED Note Section. Patient symptoms are present and not related to past medical history or a medication prescribed unrelated to the present encounter.

ED Note Section	Include	Exclude
Chief complaint	Symptom present	
History of presenting illness	Symptom present	
Review of systems	Symptom present	
Physical exam	Symptom present	
Vital signs	Fever present	
Family history		Always (e.g. brother had a cough)
Past medical history		Always (e.g. patient had cough three months ago)
Social history		Always (e.g. contacts in daycare have vomiting)
Medication list		Always (e.g. albuterol PRN for cough)
Investigations		Always (e.g. opacity on chest x-ray)
Assessment and plan, course, evaluation	Symptom present	
Final diagnosis	Symptom present	
Discharge Instructions	Symptom present	

Criteria 3: Symptom specific. Positive symptom mentions explicitly state the symptom or a predefined synonym.

Symptom specific	Include	Exclude
New loss of taste or smell	Anosmia, loss of taste, loss of smell	Injury related to loss of taste/smell
Congestion or runny nose	Rhinorrhea, congestion, discharge, nose is dripping, running, or stuffy	
Cough	Tussive or post-tussive, cough is unproductive, productive, dry, wet, or producing sputum	Wheeze, crackles, croup
Diarrhea	Diarrhea or watery stool	Loose stool, bloody stool
Fatigue	Fatigue, tired, exhausted, weary, malaise, feeling generally unwell	Looked ill
Fever or chills	Fever, pyrexia, chills, or temperature >= 100.4 °F [38 °C]	Afebrile, felt warm
Headache	HA/headache, migraine, cephalgia, head pain	Headache due to injury
Muscle or body aches	Myalgias, myoneuralgia, muscle or body aches, soreness, generalized aches and pains	Localized pain, injury, abdominal pain, lower back pain
Nausea or vomiting	Nausea, vomiting, emesis, throw up, queasy, regurgitated	Gastritis, gastroparesis
Shortness of breath or difficulty breathing	Dyspnea, breathing is short, difficult, increased, labored, or distressed	BiPAP, CPAP, oxygen need
Sore throat	Sore throat, throat pain, pharyngeal pain, pharyngitis, odynophagia	Streptococcus, dysphagia, hoarseness, red throat
