# Supplementary material for: Moving Biosurveillance Beyond Coded Data Using AI for Symptom Detection From Physician Notes: Retrospective Cohort Study
Source: J Med Internet Res. 2024 Apr 4;26:e53367. doi: 10.2196/53367 (PMC11027052; doi:10.2196/53367)
Supplement: Multimedia Appendix 2 [file jmir_v26i1e53367_app2.pdf]

Multimedia Appendix 2. Time series figures. Prevalence of 11 COVID-19 symptoms detected by natural language processing and ICD-10 coding during emergency department encounters with patients with COVID-19.

Supplementary information for:

Moving Biosurveillance Beyond Coded Data Using AI for Symptom Detection from Physician Notes: Retrospective Cohort Study

Andrew J. McMurry,[1,2] Amy R. Zipursky,[1,3] Alon Geva,[1,4,5] Karen L. Olson,[1,2] James R. Jones,[1,2] Vlad Ignatov,[1] Timothy A. Miller,[1,2] Kenneth D. Mandl[1,2,6,*]

[1] Computational Health Informatics Program, Boston Children's Hospital, Boston, Massachusetts, United States of America
[2] Department of Pediatrics, Harvard Medical School, Boston, Massachusetts, United States of America
[3] Division of Emergency Medicine, Department of Pediatrics, The Hospital for Sick Children, Toronto, Ontario, Canada
[4] Division of Critical Care Medicine, Department of Anesthesiology, Critical Care, and Pain Medicine, Boston Children's Hospital, Boston, Massachusetts, United States of America
[5] Department of Anaesthesia, Harvard Medical School, Boston, MA, USA
[6] Department of Biomedical Informatics, Harvard Medical School, Boston, Massachusetts, United States of America

*Corresponding author
E-mail: kenneth_mandl@harvard.edu (KDM)

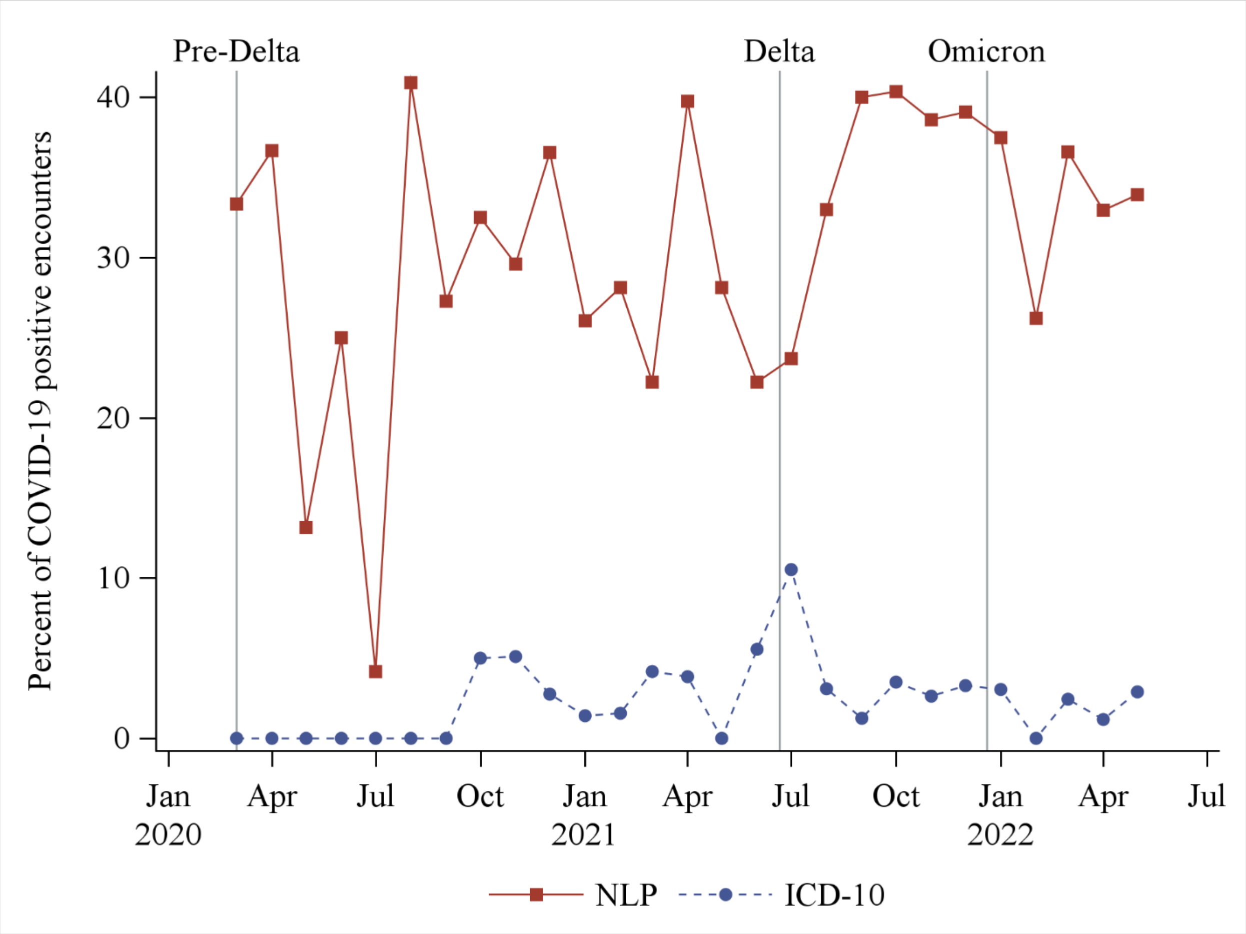

Figure S1. Percent of encounters with patients with COVID-19 presenting to the emergency department each month with **congestion or runny nose**, as measured using NLP and ICD-10.

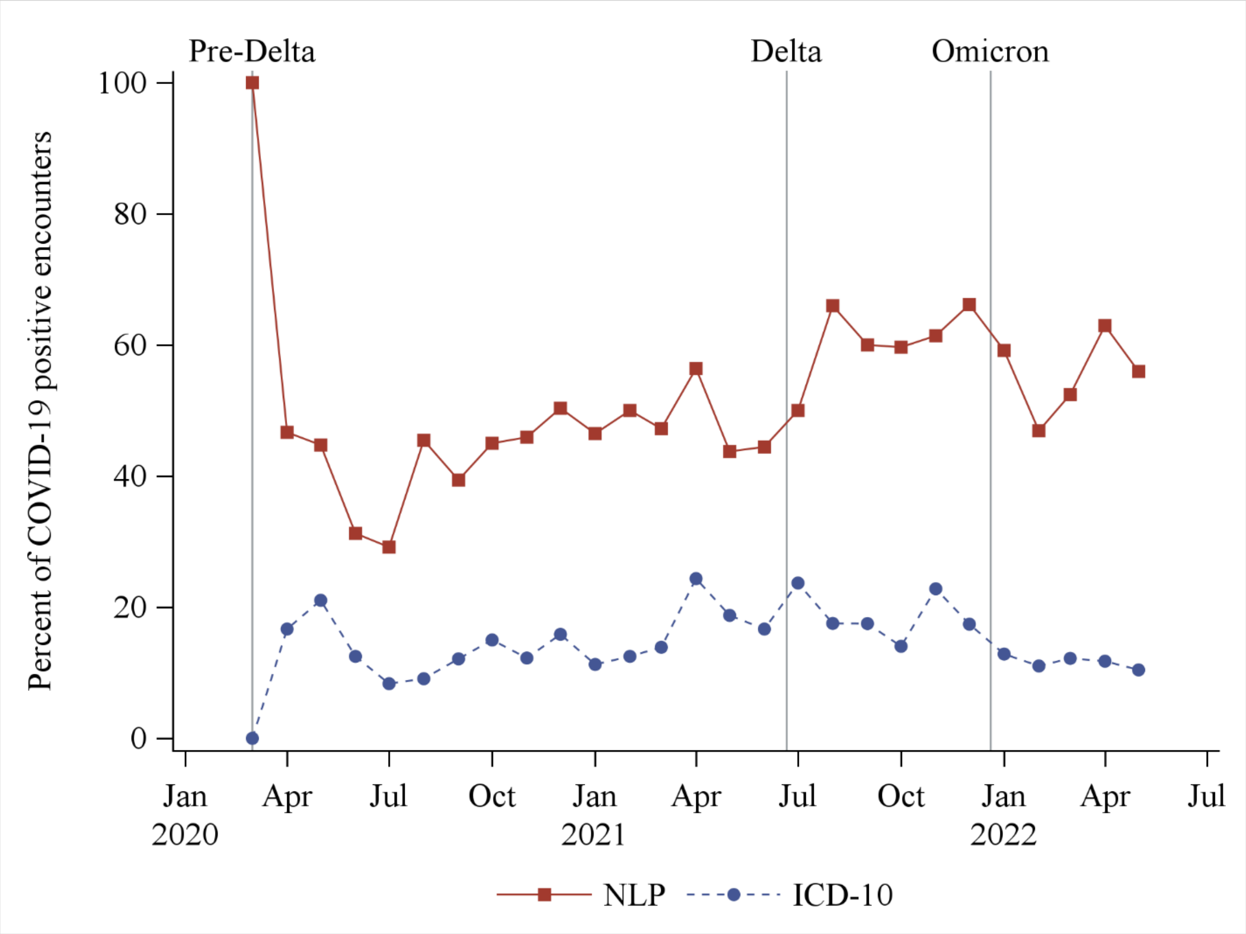

Figure S2. Percent of encounters with patients with COVID-19 presenting to the emergency department each month with **cough**, as measured using NLP and ICD-10.

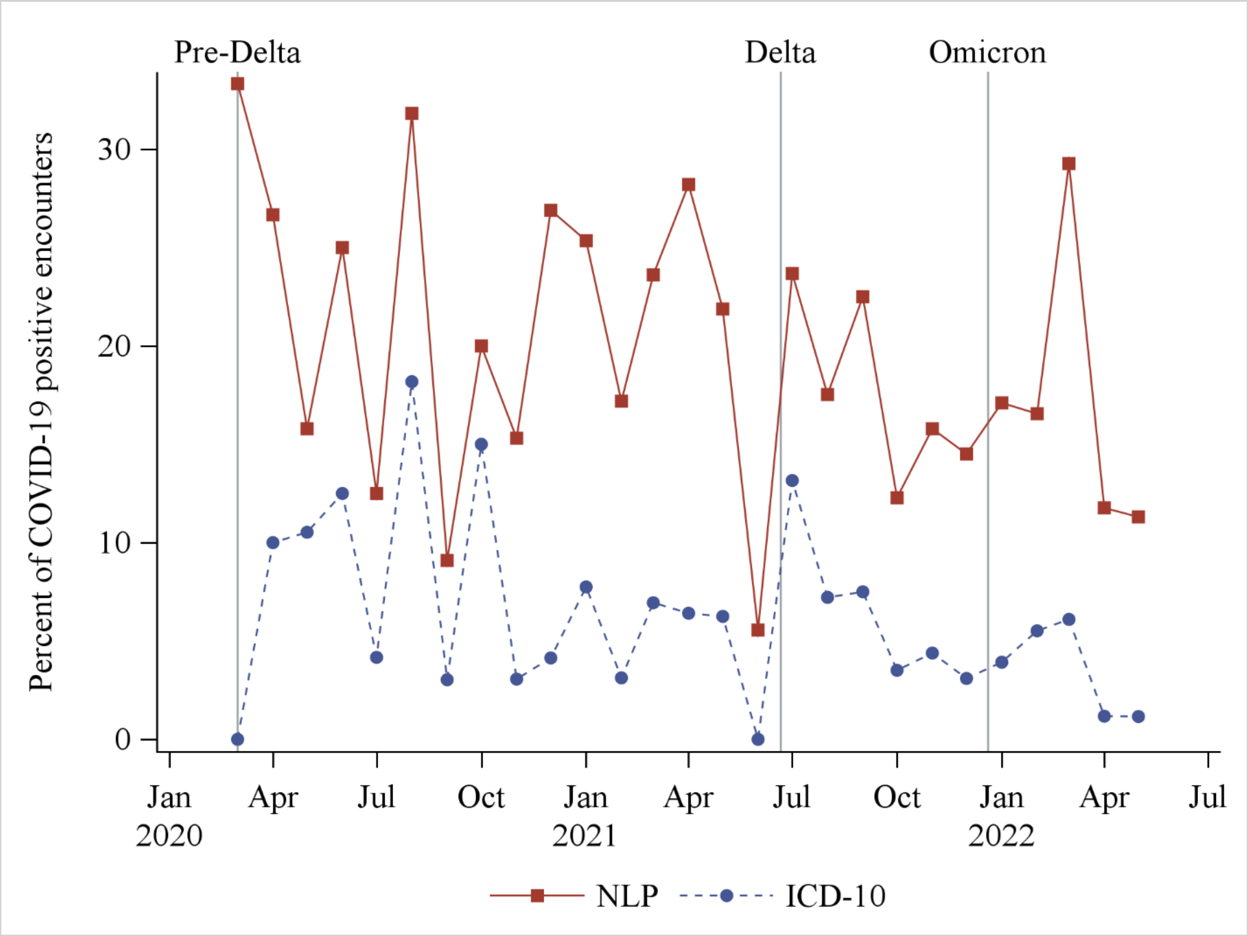

Figure S3. Percent of encounters with patients with COVID-19 presenting to the emergency department each month with **diarrhea**, as measured using NLP and ICD-10.

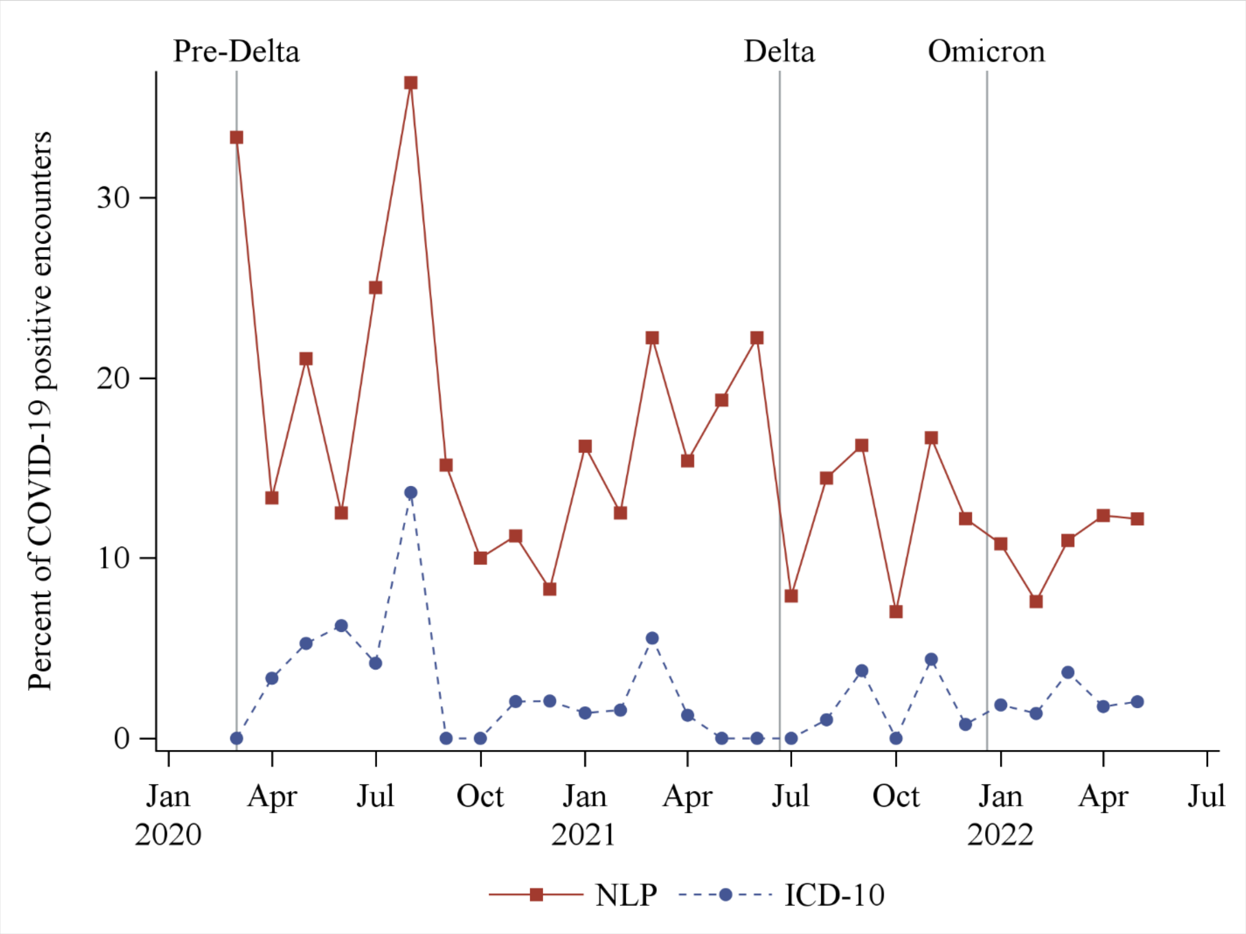

Figure S4. Percent of encounters with patients with COVID-19 presenting to the emergency department each month with **fatigue**, as measured using NLP and ICD-10.

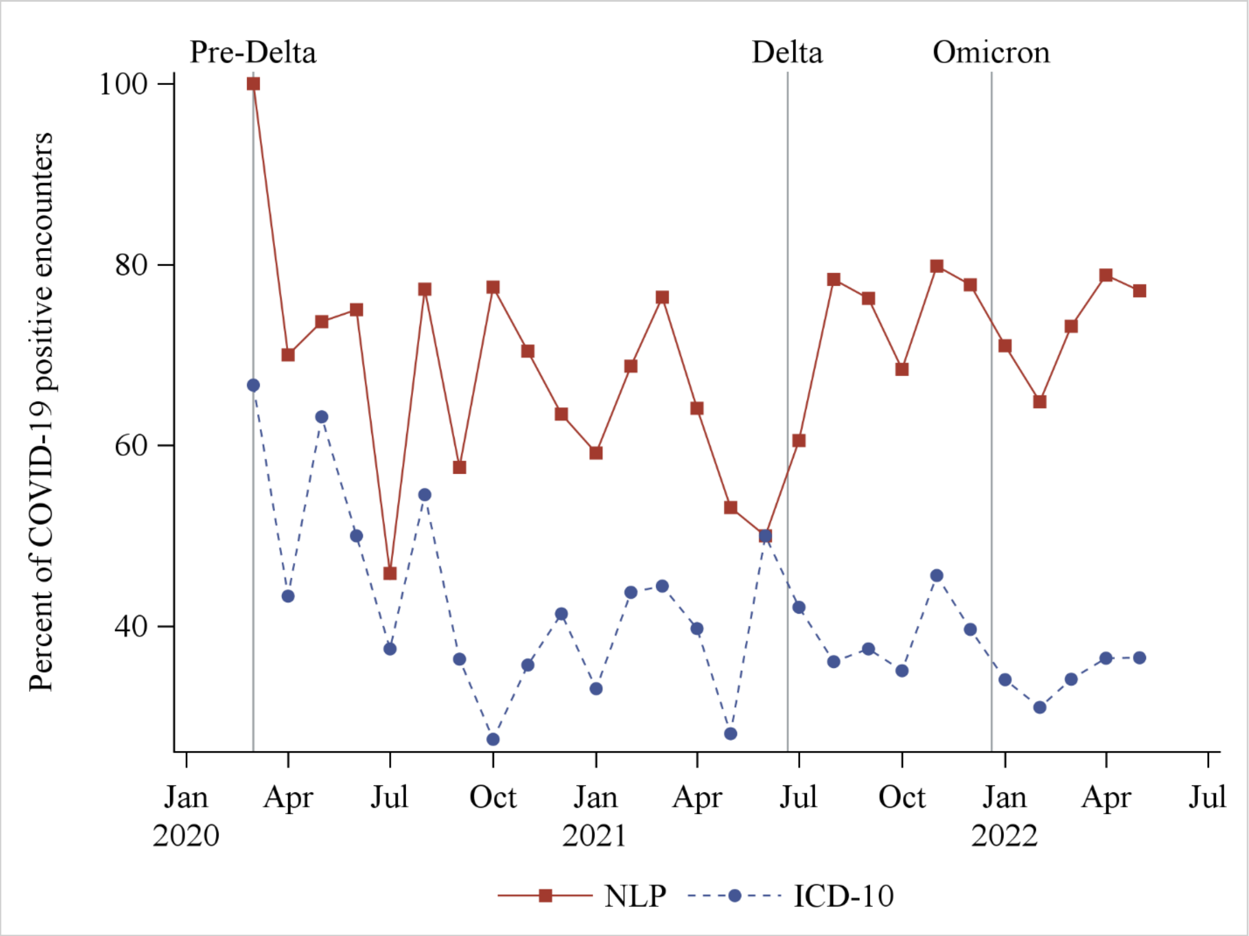

Figure S5. Percent of encounters with patients with COVID-19 presenting to the emergency department each month with **fever or chills**, as measured using NLP and ICD-10.

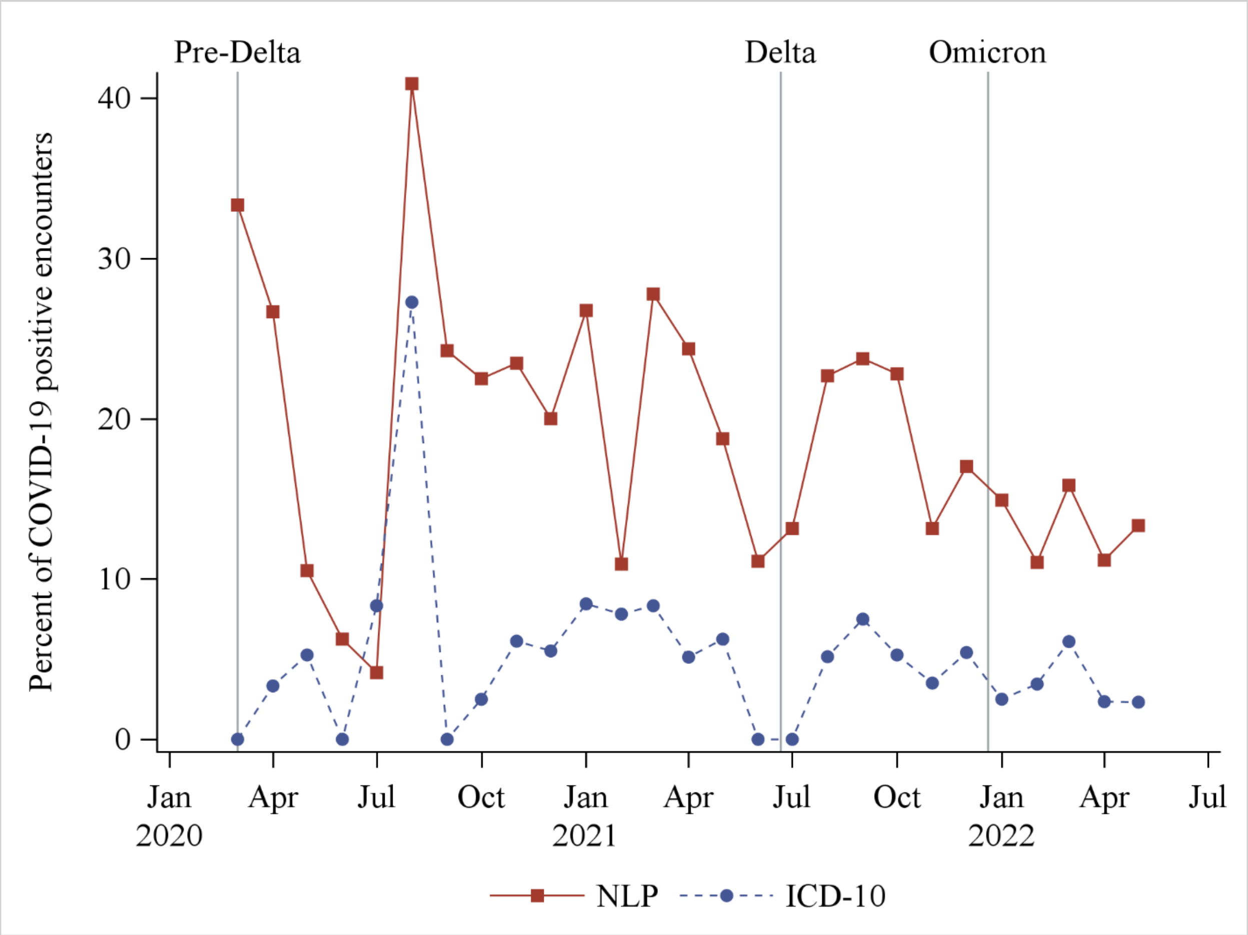

Figure S6. Percent of encounters with patients with COVID-19 presenting to the emergency department each month with **headache**, as measured using NLP and ICD-10.

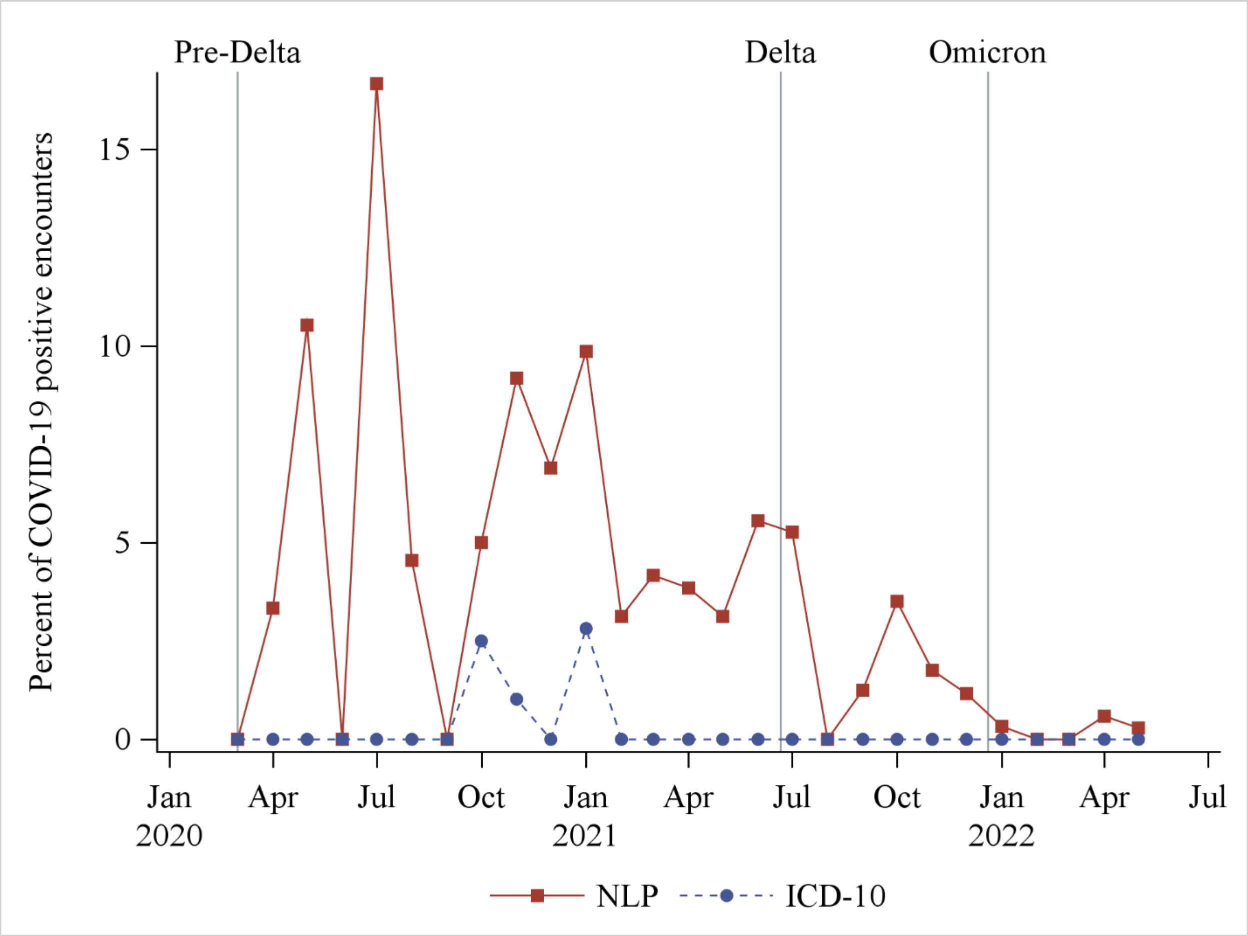

Figure S7. Percent of encounters with patients with COVID-19 presenting to the emergency department each month with **loss of taste or smell**, as measured using NLP and ICD-10.

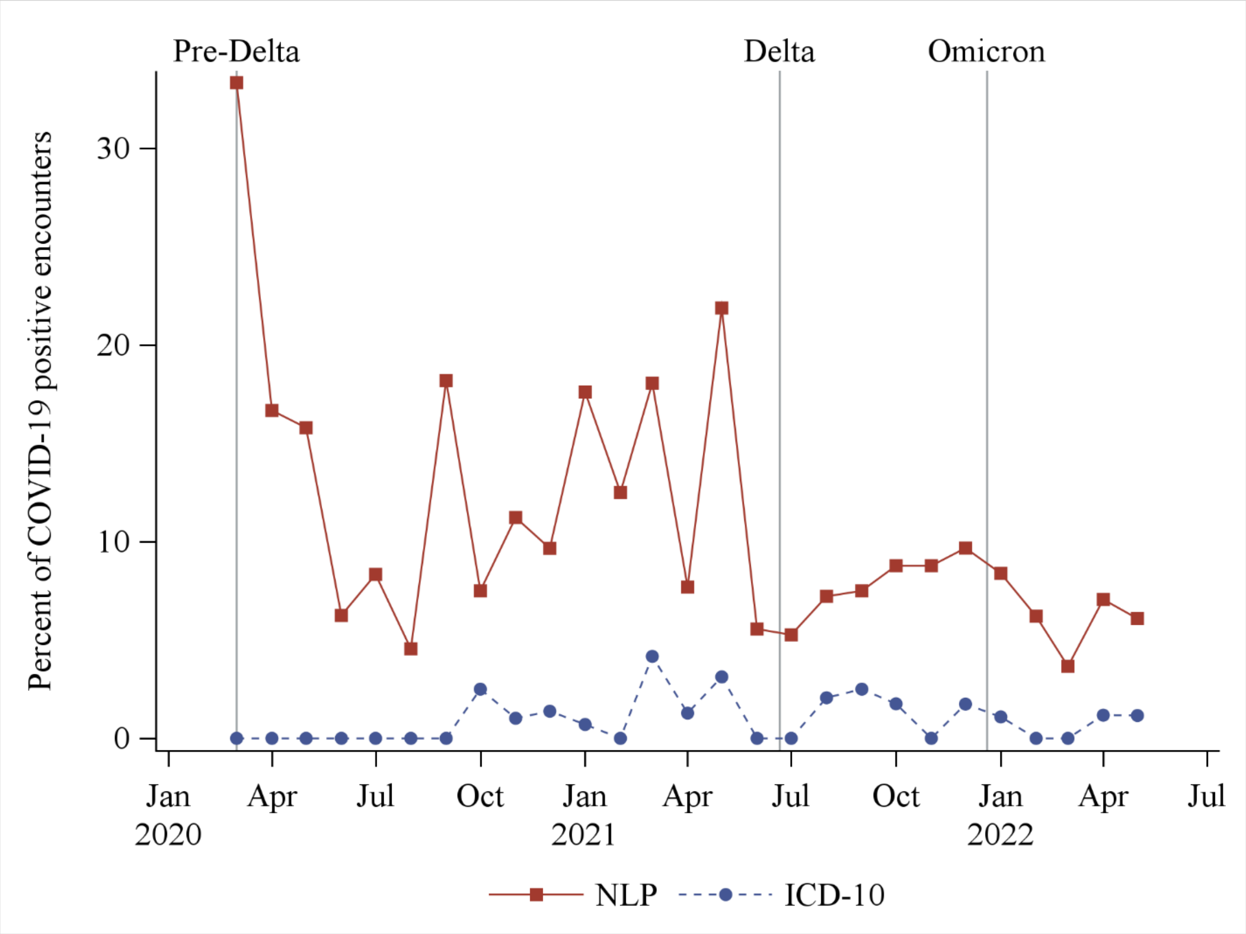

Figure S8. Percent of encounters with patients with COVID-19 presenting to the emergency department each month with **muscle or body aches**, as measured using NLP and ICD-10.

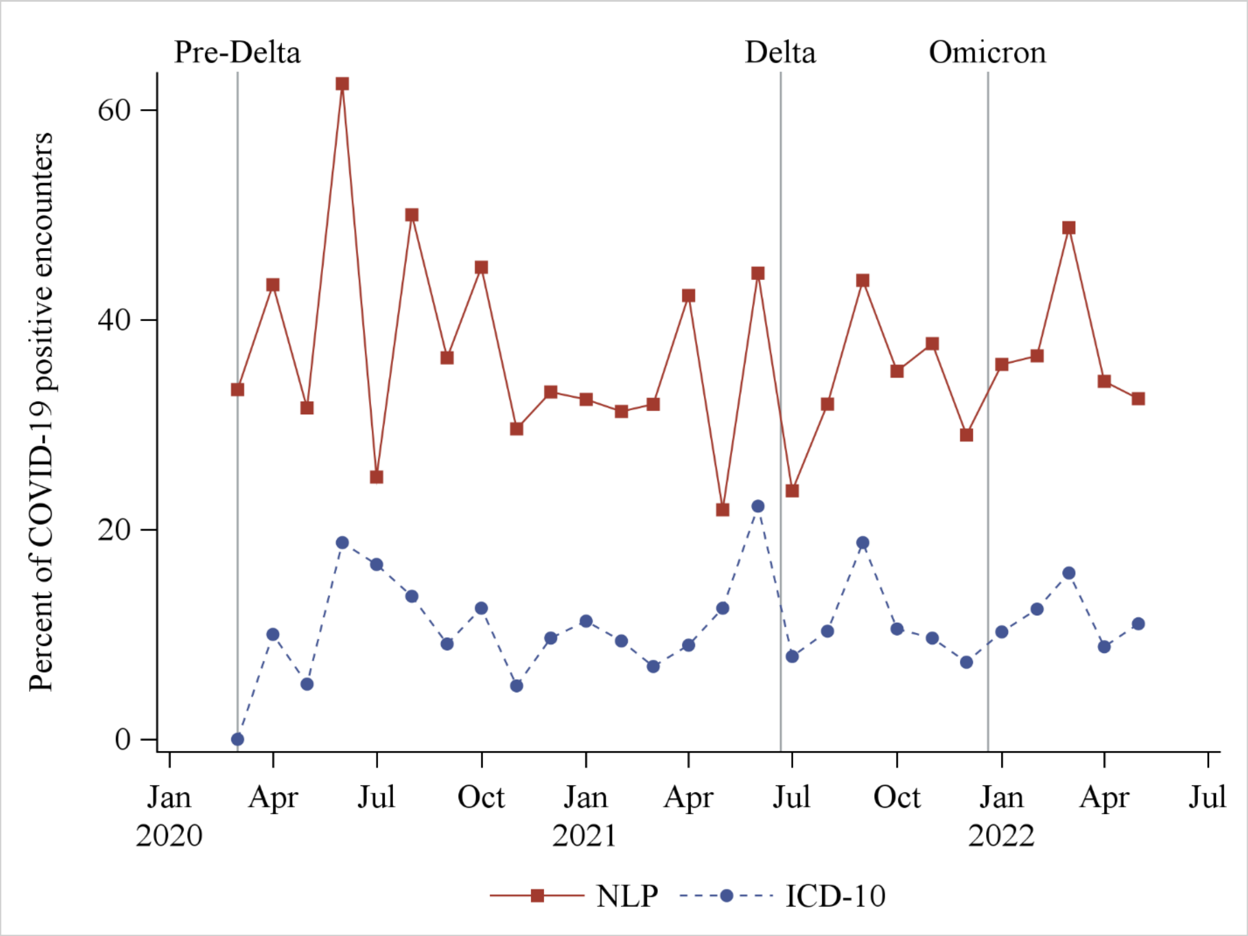

Figure S9. Percent of encounters with patients with COVID-19 presenting to the emergency department each month with **nausea or vomiting**, as measured using NLP and ICD-10.

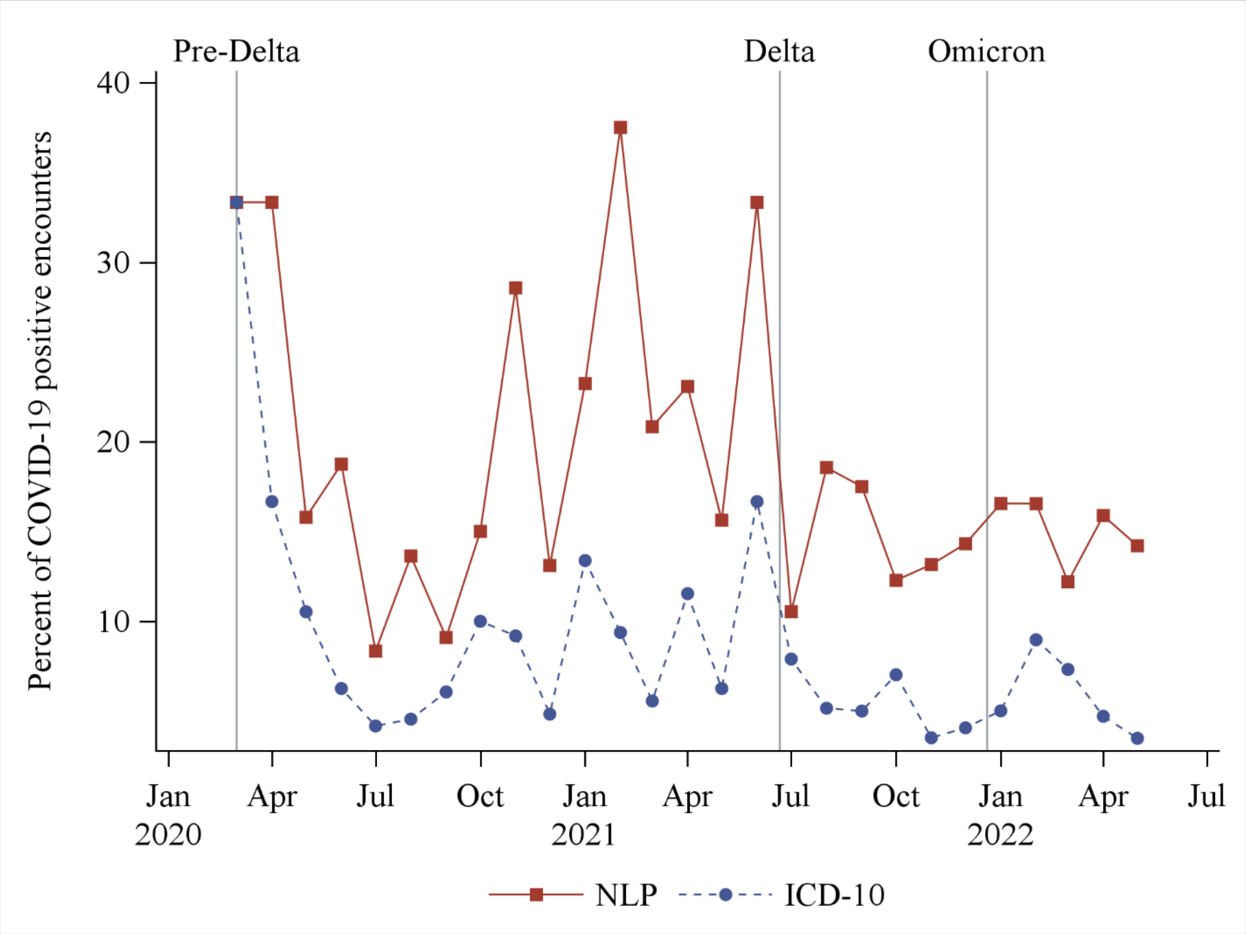

Figure S10. Percent of encounters with patients with COVID-19 presenting to the emergency department each month with **shortness of breath or difficulty breathing**, as measured using NLP and ICD-10.

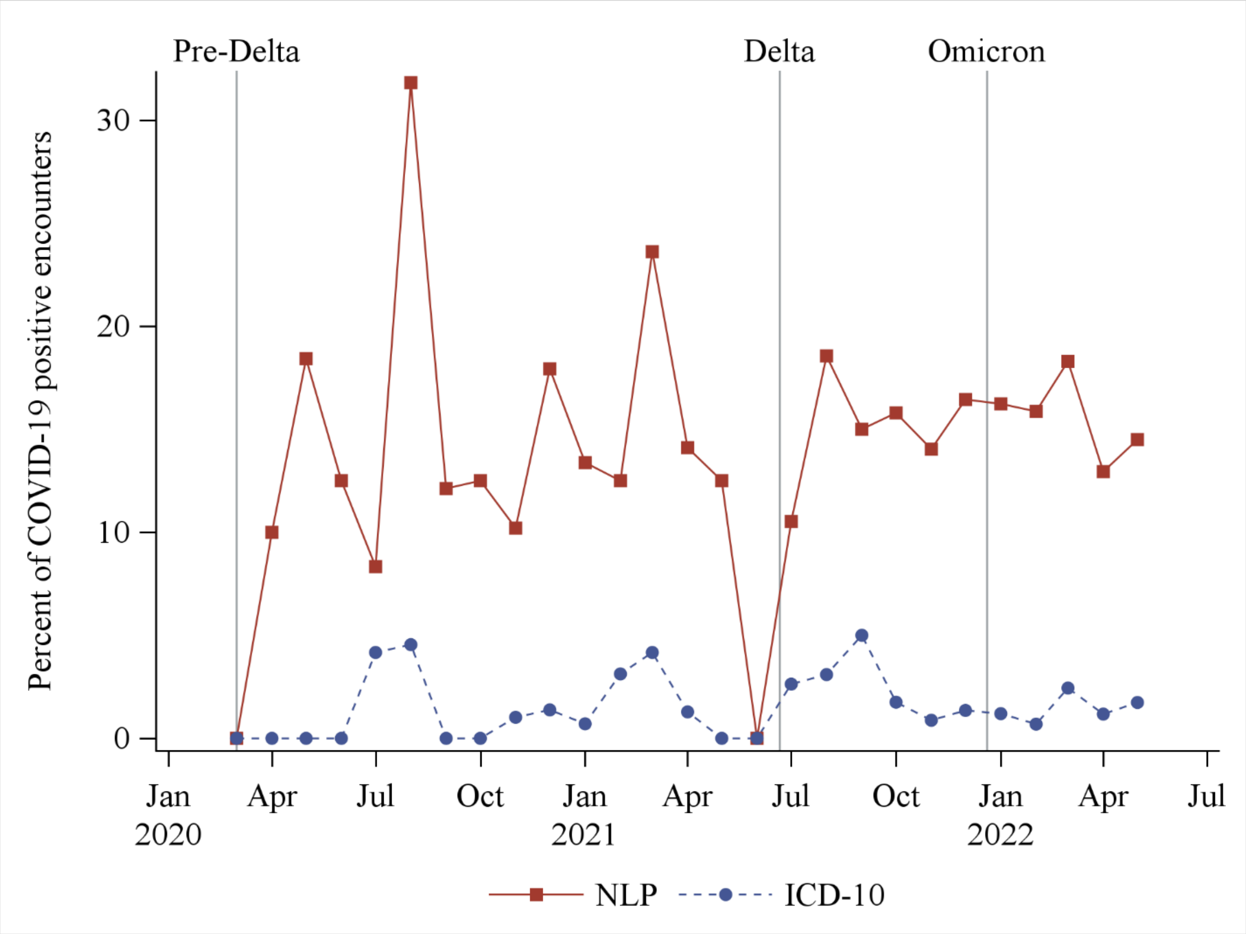

Figure S11. Percent of encounters with patients with COVID-19 presenting to the emergency department each month with **sore throat**, as measured using NLP and ICD-10.
